# Resilience and prosocial behaviour in Spanish care professionals of dependent persons

**DOI:** 10.1111/hsc.14110

**Published:** 2022-11-22

**Authors:** Manuel Martí‐Vilar, Alba Carreras Tortosa, Alicia Sales‐Galán, Guido Corradi

**Affiliations:** ^1^ Faculty of Psychology and Speech Therapy Universitat de València València Spain; ^2^ Department of Psychology, Faculty of Education and Health Camilo José Cela University Madrid Spain

**Keywords:** health care professionals, mental health, nursing homes, older people, prosocial behaviour, resilience

## Abstract

Professional residential care providers face several stressors due to the burden of caring for dependent people. This burden may affect the way in which care is carried out. Resilience, as personal strengths, may help them to be more effective in their workplace and in their interaction with patients, and this may be related to the development of participation skills and prosocial behaviours. A total of 125 professional's caregivers from Spain responded to the Connor‐Davidson Resilience Scale Resilience Scale and the PB new Prosocial Conduct Scale over the years 2018 and 2019. We checked the predictive power of resilience as well as other predictors (sex, type of contract and total months worked in professional caregiving) on prosocial behaviour in caregivers' professionals with multiple regression analysis. Results showed resilience as the only significant predictor, explaining 21% of the variance in prosocial behaviour (*R* ^2^ = 0.21, *F*(5, 115) = 6.16, *p* < 0.001). This indicates that resilience is a variable prediction of prosocial behaviour in health and social professionals. Resilience gets in the individual the capacity to be attentive to give answers in certain situations, being a predictor of great relevance of the prosocial behaviours. Thus, it is necessary to deepen the research on professional caregivers to be able to train and empower them in skills that improve their quality of life and by strength, that of dependent people.

**What is known about this topic**

- Previous studies have reported that the situations of dependency as care of people with dementia carried to burden on professional caregivers of nursing homes.
- There is less emotional exhaustion and greater efficiency and work commitment in those professional caregivers who have higher levels of resilience.
- Some authors state that having resilient behaviours increases the probability of overcoming obstacles and being attentive to give answers in certain situations, being a very relevant predictor of prosocial behaviours.

**What this paper adds**

- More resilient professional caregivers have more interaction and involvement with patients by developing prosocial behaviour.
- The application of personal or communication skills training to caregivers of people with dementia can be considered an essential part of care.
- This quantitative study provides new lines of research on the importance of prosocial behaviour and resilience in care professionals.

## 1 INTRODUCTION

Thanks to the improvement in health and quality of life, life expectancy and the demographic growth of the aging population have been increasing. According to data from the Continuous Register (INE), life expectancy in Spain in 2020 averaged 85 years for women and 70.6 for men (INE, 2020). Recent studies (Abellán et al., 2018) indicate that Spain continues with the predicted aging process with 8,764,204 elderly persons (65 years of age and over), that is, 18.8% of the total population (46,572,132).

Continual growth in the elderly population has been accompanied by a constant growth in neurocognitive diseases, one of the most common illnesses in this population group (World Health Organization, 2018). These diseases are characterised by a progressive and irreversible deterioration of cognitive abilities, especially memory. The deterioration of such capacities can cause a situation of dependence, which is why they are considered the leading cause of disability in the elderly population (Reymond et al., 2019).

Other particularly stressful aspects are the behavioural and psychological symptoms of dementia, which further aggravate the situation of dependency, having a great impact as they do both on patients themselves and on their caregivers. They can increase the latter's feeling of being overloaded with carework (Losada et al., 2017), whether they are professionals or family (Kales et al., 2015). For this reason, overloading caregivers with work produces an increased likelihood of use of social resources or services such as institutionalisation (Ismail et al., 2016). Still, several studies indicate that these symptoms are often a source of stress and anguish, even for professional caregivers (Cerejeira et al., 2012; Fauth & Gibbons, 2014; Moore et al., 2013; Ornstein et al., 2013).

Care professionals working in residences and day centres include social work professionals, psychologists, nurses and nursing assistants, doctors, occupational therapists, physiotherapists, sociocultural facilitators, administrators and others. That is, they are those people who carry out tasks whose main purpose is to promote health, as well as to care for it and cure pathologies or diseases (Rodríguez & Ortunio, 2019). Such professionals are subjected to a series of stressors, both in the work and personal spheres, which can affect them emotionally, generating negative consequences in their personal adjustment to their working environment, and affecting the way in which they take care of dependent elderly people (Torres, 2008).

Various studies have shown that there is a close relationship between the resilience of caregivers and variables such as the context in which the work of care is performed, the attitude of the person receiving the care, the resources (professional and informal) of the caregiver, and the feedback that the caregiver receives for their services (Gaugler et al., 2007). In turn, it has been shown that caregivers who have more flexibility in certain adverse situations are more resistant than those with low recovery capacity (Menezes de Lucena et al., 2006), so that resilience is a fundamental pillar in the effective self‐care of the professional caregiver. This is related to what Forján and Morelatto (2018) found: in their study, the group of teachers working in vulnerable contexts more commonly showed medium or high levels of factors such as “significant participation” and “prosocial behaviour.”

Nevertheless, not all caregivers feel trained or qualified to give proper caring for the elders. According to Izal et al. (2003), just 5.5 out of 10 workers feel competent to manage behavioural problems or daily challenges. These data reflect the importance of personal skills training.

An increased number of authors emphasise the need for professional caregivers to have optimal knowledge to carry out their work and to better cope with the most complex adversities and situations (Farran et al., 2007; Huang et al., 2015; Tan et al., 2017; Teri et al., 2005). They need to have continuous and qualified training in technical skills, communication skills, and affective‐relational aspects. Nonetheless, a recent review by Kong et al. (2017) concluded this training is insufficient in care centres. Training of professionals in the care sector, focus exclusively on the health‐related aspects of their work, forgetting the need to develop general interpersonal skills, such as communication, which favour the establishment of a positive and trusting relationship with the caregiver and their families (Roche & Escotorin, 2017), in addition to specific skills for the management of Prosocial Behaviour Scale (PBS; Cohen‐Mansfield et al., 2015).

The research carried out on professional care highlights caregivers' experience of a burden, and more specifically, chronic work‐related stress or burnout syndrome, since caregivers often show emotional and physical signs of chronic stress (Flores et al., 2014). Yıldızhan et al. (2019) wrote of “staff exhaustion”, referring to a phenomenon of stress or overburdening, depersonalization (staff can be recalcitrant towards the positive aspects of the people with whom they interact), and feelings of personal failure. Several environmental factors may contribute to this situation, such as constantly doing the same job with the same type of patients, patients showing slow progress, excessive work overload, lack of reward in the workplace, etc (Taycan et al., 2013).

Several studies have shown that there is a close relationship between the resilience of caregivers and variables including the context in which the carework is performed, the attitude of the person receiving the care, the resources (professional and informal) of the caregiver, and the feedback that the caregiver receives for their services (Gaugler et al., 2007). For Connor and Davidson (2003), resilience is the set of qualities, resources or strengths that enable individuals to progress by successfully coping with adversity.

In health professionals, the resilience variable is related to personality, through a series of traits that serve to improve the functioning and well‐being of these professionals, and it is objective, that the improvement in building one's resilience can help to reduce stress, as well as the impact that work can have on the person, in order to improve their physical and mental well‐being (García, 2014).

The type of contract and the length of time worked could also have an impact. The effect of stressors may differ depending on the type of contract and the professional category. Geriatric nurses and assistants may be the most affected jobs by stressors (who represent the majority of the workforce). They often complain about performing less rewarding and less valued activities without proper objectives, receiving more supervision than information or participation in decision making, and their promotion limitations due to their low specialisation (González & Domínguez, 2000).

Concerning the time spent working, developing the same type of work with the same patients over a long period of time can be exhausting and decrease personal effectiveness. Developing strategies is necessary to combat these stressful effects and may lead to greater resilience (Judkins, 2001). Therefore, age and years of work may suggest a relation with stress. This idea is consistent with Mollart, Skinner, Newing and Foureur's study (2013), who found that those who have been working less time are more likely to develop Burnout Syndrome. However, other studies report the opposite results, showing that older caregivers perceive higher stress (Sarabia‐Cobo et al., 2016; Torres, 2008). In his review, Torres (2008) defends that the coexistence of risk and protective factors of stress may explain this ambiguity. Some stressing factors could be professional immaturity and time pressure at work and some protective variables could be learning motivation and self‐confidence.

Gender is another relevant issue. Historically, society has attributed women to the role of caring, arguing they have better skills for it (Rodríguez, 2015). Currently, tasks of care continue to be predominantly female due to the fact these jobs are mainly performed by women (Lago & Alós, 2012; Sanz et al., 2016). Likewise, several studies show that there is a sex difference in stress and symptoms and that women present higher levels of stress than men (Ramírez & Hernández, 2008). In another study, Martín (2015) identified staff shortages as a labor stress factor for 92.4% (92 sample persons), and it affected more women than men (92.3% vs. 80%).

Related to sex and resilience, some studies argue that women score higher on the external protective factor, that is, they require more external support at different ages, compared to men who score higher on the internal protective factor (González‐Arratia & Valdez, 2013).

Regarding sex and resilience, some studies argue that women score higher on the external protective factor compared to men, who score higher on the internal protective factor (González‐Arratia & Valdez, 2013). However, studies that have considered both sex and resilience present disparate results, finding higher levels of resilience in women and no differences by gender. Therefore, further empirical research is needed to provide accurate conclusions about this relationship (González‐Arratia & Valdez, 2013).

However, not everything is negative. Positive characteristics of the caregiver work as a protective factor, such as empathy, self‐esteem, resilience, problem‐centred coping, social support, or satisfaction (Ruiz‐Robledillo & Moya‐Albiol, 2012). These factors hinder the development of stress and its negative consequences on health.

Menezes de Lucena et al. (2006) showed that there was less emotional exhaustion and greater work efficiency and commitment in those professional caregivers who had higher levels of resilience. This concept refers to the set of qualities, resources or strengths that favour individuals making successful progress when they face adversity according Connor and Davidson (2003).

García (2016), studied the application of a resilience‐based approach in the professional field, which has provided evidence of its relationship to other variables associated with the work context, giving rise to programs for the promotion of resilience based on training, that cover working life, health and wellbeing, and personal relationships. In this way, resilience can be considered as a key feature of a successful professional; that is, individual resilience levels can determine who is relatively successful and who fails in the competitive dynamics of the contemporary workplace (Rook et al., 2018).

Specifically, with regard to the work of professional caregivers of institutionalised older persons, as indicated by Menezes de Lucena et al. (2006) resilience can help such caregivers to be more effective in their job and in their interaction with patients, since it is not that they do not feel overloaded by their work, but that they develop better skills at participating in their work and bearing the burden. Indeed, some authors state that having resilient behaviours increases the likelihood that an individual overcomes the obstacles that he or she confronts every day. Drawing on resilience, an individual can develop higher levels of prosocial behaviour (Crespo et al., 2010; González et al., 2008). That is, an individual's resilience grants her or him the ability to ready with appropriate responses for particular situations, and is especially relevant in determining their readiness to respond with prosocial behaviours.

In this context, as Auné et al. (2014), indicate, it is highly relevant to consider care professionals' tendencies towards prosocial behaviour. Some authors Martí‐Vilar and Navarro (2017) define prosocial behaviour as those observable behaviours that can benefit or generate positive consequences in others; or, in other words, are behaviours that have to do with help, cooperation, and/or solidarity. According to these authors, prosocial behaviours imply a certain degree of understanding about what happens to the other or about the needs they have; that is, putting oneself in another's shoes. Martorell et al. (2011) describe prosocial behaviour as a voluntary behaviour that benefits others; that is linked to emotional development and personality and that includes actions of help, cooperation and altruism.

Likewise, Plaza‐Carmona and Requena‐Hernández (2016) highlighted the importance of generating policies that strengthen the professional profile of a health worker as a means to encourage proactive health‐related behaviours towards users, so that in the long term they achieve greater wellbeing, leading to economic, social and health savings, especially in the elderly people. Thus, it can be shown that health professionals' prosocial behaviour is related to enhanced wellbeing in dependent elderly people in a way that nurtures positive care processes. More generally, within the social sciences in recent years, interest in the study of prosocial behaviour has increased (Escotorin & Cirera, 2013). In the field of psychology, important progress has been made on how prosocial behaviour is related to other newer variables or in the construction of instruments for its evaluation (Auné et al., 2014).

Although interest in studying prosocial behaviour has increased substantially, there are few studies that relate the construct to the work of the caregiver of dependent persons, especially in regard to professional caregivers. While it is true that there are articles that highlight the importance of prosocial behaviour in creating relationships of trust and in strengthening the thoughts, attitudes and behaviours that underpin caregivers' work (Escotorin & Cirera, 2013), this idea has not been elaborated on in more detail in relation to caregivers' actual working practices or their qualities of resilience.

In this sense, Abraha et al. (2017) reiterate that professional or family caregivers have the strongest impact on the wellbeing of people with dementia. The success of the interventions depends largely on caregivers' collaboration. Due to its essential role, it is crucial to evaluate some of their psychological variables, as their attitude directly influences on dependent people's care.

Hence, this paper aims to analyse the relationship between resilience, some sociodemographic aspects (sex, type of contract, and total months worked in professional caregiving) and prosocial behaviour in professional caregivers of people in dependent situations. The specific objective is to analyse whether resilience and sociodemogrphic aspects is a predictor to prosocial behaviour in such professionals.

## 2 METHOD

### 2.1 Participants

The sample consisted of 125 professional caregivers from different public care elderly centres in the Valencian region, Spain. The sample was collected thanks to the government authorities of the region, who helped with asking for permissions and giving access to the public centres. After that, the centres included were randomised. Also, participants within each centre were recruited through non‐probabilistic convenience sampling. The sample was recruited in 2019, between the months of July and December, prior to the declaration of the alarm state because of the COVID‐19.

The sample's mean age was 42.87 years (SD = 13.49), and it was composed of 86.7% women and 13.3% men. 16.7% had primary school studies as their highest qualification, 33.3% secondary school studies and 50% university‐level studies.

In relation to the type of centre in which they worked, it was observed that 56.7% of the professionals worked in a residence and day centre, 30% in a residence and 13.3% in an association. Of this sample, 23.3% were nurses, 60% nursing assistants and 16.7% psychologists. The inclusion criteria were those professionals who were working in a care centre for elderly people in situations of dependency, who had a full‐time contract and who had been working there for at least 36 months. The average number of months worked was 128.97 (SD = 60.61). Those who, despite their contract and length of tenure, did not have direct formal contact with patients (such as cleaning staff or cooks) were excluded from the sample.

### 2.2 Instruments

First, the following sociodemographic variables were evaluated in caregivers: sex, age, educational level, professional role, workplace, sector, months of experience as a caregiver for people in situations of dependency and type of contract. Additionally, questionnaires on resilience and prosocial behaviour were completed. To measure resilience, the Connor‐Davidson Resilience Scale Resilience Scale (Connor & Davidson, 2003) was used, in its abbreviated and validated version for the Spanish population introduced by Notario‐Pacheco et al. (2011). This version consists of 10 items. In this scale, participants are asked to respond to what extent they agree with each of the phrases presented to them (for example, item 1: “I am able to adapt to changes”). The response form uses Likert scales of 5 points from 0 (totally disagree) to 4 (totally agree). To measure prosocial behaviour, the PB‐NEW Prosocial Behaviour Scale was used Caprara et al. (2005) which is a scale of 16 items, each with 5 Likert‐type response options. Each item reflects behaviours and feelings that could be classified as one of four kinds of actions: helping, sharing, caring or showing empathy to others.

### 2.3 Procedure

Once the government permissions were obtained and the access to the centres was allowed, the people in charge of the management of the institutions were contacted in order to request their authorization to evaluate members of their staff, informing them of the inclusion criteria and the objectives of the research. In parallel, approval was requested from the Ethics Commission of the University of Valencia, and after receiving both types of authorization, we proceeded with the study. In this way, those who wanted to participate voluntarily and who met the criteria, first signed an informed consent form and then answered the questions, which being a self‐report measure they could answer in their free time after finishing the working day, with an approximate duration of 20 min. Thus, a room was set up for them to respond properly. A psychologist researcher was present in this room, in order to assist them and solve the questions that may arise while answering the questionnaires. Also, the anonymity was ensured. After that, subjects deposited their answers in a box to preserve their anonymity.

### 2.4 Analysis

We report descriptive statistics for the different groups of relevance. We inspected correlation between variables. Also, for inferential purposes we performed a regression analysis was carried out using jamovi open‐source software The jamovi Project, (The jamovi project, 2022) with *Total Prosocial Behavior* as the outcome variable, predicted by the *CD Resilience Total* as well as other predictors such as sex, type of contract, and total months worked in professional caregiving.

## 3 RESULTS

Descriptive statistics showed no differences between occupational status (see Table 1) and psychological variables of interest. Means for CDTotal ranged from 28.3 and 35.3 from a potential range of 0 to 40, while CProsTotal means ranged from 38 to 56.3 from a potential range of 16 to 80.

**TABLE 1:** Descriptives

Workstation	Connor‐Davidson resilience total	Prosocial behaviour total
*N*		
Nursey	12	12
Social worker	3	3
Occupational therapist	1	1
Nursing assistant	81	80
Psychologist	12	12
Physiotherapist	4	4
Others	11	10
*M*		
Nursey	30.5	48.8
Social worker	28.3	55.3
Occupational therapist	30.0	38.0
Nursing assistant	32.7	52.3
Psychologist	32.4	52.2
Physiotherapist	35.3	52.0
Others	34.1	56.3
SD		
Nursey	5.65	6.73
Social worker	3.51	7.02
Nursing assistant	5.02	7.33
Psychologist	3.12	5.49
Physiotherapist	3.77	4.24
Others		

Correlation analysis reveal a moderate to high correlation between CDTotal and CProscTotal (see Table 2). We did not find any significant correlation between age and months worked.

**TABLE 2:** Correlation among variables

	Age	Months working	Connor‐Davidson total	Prosocial behaviour total
Age				
*R*	—			
*p*	—			
Months working				
*R*	0.32	—		
*p*	<0.001	—		
CDTotal				
*R*	0.05	0.01	—	
*p*	0.526	0.898	—	
ProscBTotal				
*R*	0.06	−0.04	0.44	—
*p*	0.506	0.640	<0.001	—

The results show (see Table 3) that the model explains 21% of the variance in prosocial behaviour (*R* ^2^ = 0.21, *F*(5, 115) = 6.16, *p* < 0.001), with resilience score as the only significant predictor. This indicates that those people who are resilient will exhibit more prosocial behaviours. There was a positive effect of the resilience variable (*β* = 0.66, 95% CI [0.41; 0.90], *p* < 0.001), while the other variables did not have statistically significant effects (see Tables 1).

**TABLE 3:** Model regression coefficients

Predictor	Estimate	SE	95% confidence interval		*t*	*p*	Stand. estimate	95% confidence interval	
			Lower	Upper				Lower	Upper
Intercept	3.115.206	422.147	227.894	395.148	7.379	< 0.001			
CDTotal	0.65833	0.12329	0.4141	0.9026	5.340	< 0.001	0.44745	0.2815	0.0435
Sex									
Female—male	139.200	173.593	−20.469	48.309	0.802	0.424	0.06707	−0.0986	0.3776
Contract									
Temporary—permanent	116.038	181.655	−24.382	47.589	0.639	0.524	0.09070	−0.1906	492.601
Other—permanent	0.85283	255.499	−42.086	59.142	0.334	0.739	882.770	−435.632	40.197
Tenure in job	−0.00406	0.00850	−0.0209	0.0128	−0.477	0.634	−0.00276	−0.0142	6.16 e‐4

**FIGURE 1:**
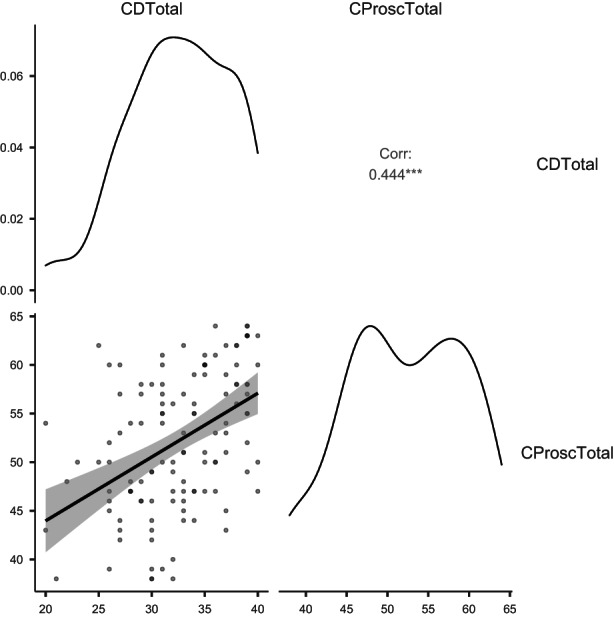
Graph of variable densities, with scatterplot showing correlation between the two variables of resilience and prosocial behaviour

Likewise, a significant and positive correlation (*r* = 0.44, *p* < 0.001) was found between the main variables of resilience and prosocial behaviour. The residuals were explored with quartile‐to‐‐quartile graphs to check their normality (see Figure 1). Likewise, the Durbin‐Watson statistics had a value considered acceptable (*d* = 1.87, *p* = 0.39) indicating that there was not too much autocorrelation between the variables.

## 4 DISCUSSION

The demographic and social changes that society is experiencing make care resources, such as nursing homes, more necessary than ever (Koopman et al., 2020). Besides, a high percentage of these institutionalised senior people have some neurodegenerative diseases. The progression and the cognitive and behavioural characterisation of these diseases (Ismail et al., 2016) leads to a worse situation of dependency. Attending to people with different neurodegenerative diseases can generate stress due to the demand and overload of high‐quality and personalised attention. Overburdening in caregivers could negatively affect them personally and professionally, harming their work performance in their work environment and their tasks of care (Torres, 2008). Therefore, recent studies focused on professional caregivers have been closely related to the negative emotions and burnout in caregivers.

The objective of this work is a change to a more positive approach, based on the study of capabilities and strengths that act as protective factors against prolonged stress, such as resilience. Such strengths can help them to increase their work effectiveness and resolution and improve their interaction with patients. Moreover, this may be related to the development of participation skills and pro‐social behaviours.

In accordance with the main hypothesis of the present study, results indicated that resilience is positively correlated with prosocial behaviour in the group of professionals that work with people in situations of dependence. Even after accounting for several other variables to do with the particular type of work that they have, resilience can be considered as a predictor of prosocial behaviour in professionals. Furthermore, the absence of predictive results considering the type of contract and time being working may be due to researchers' contradictions already mentioned in the introduction. First, while some authors consider a veteran work experience could result in stress tolerance and resilience, others uphold an increased stress perception. Second, personality and personal experiences may be more important than work experience in developing effective strategies for stress and social and emotional sensitivity to patients. Despite the influence of work characteristics and social support, it seems that the factors most strongly associated with resilience are the caregiver's intrinsic factors, such as self‐esteem, self‐efficacy or perceived ability (Fernández‐Lansac & Crespo, 2011). In addition, caregivers generally exhibit moderate‐to‐high levels of resilience and resistance, although high levels do not imply the absence of burden or stress (Fernández‐Lansac & Crespo, 2011; Wilks & Vonk, 2008). Therefore, it is possible that work characteristics do not predict prosocial behaviours since personal characteristics would be more influential than work profile ones.

Several researchers conceive resilience as the combination of individual, family, and social factors, as well as a protective function comprised of personal and social resources (Becoña, 2006).

The importance of resilience for caregivers of dependent people is clear given that such professionals face high levels of responsibility and stress in their daily work. Some authors, such as Burgueño (2008), state that direct care staff is the group most likely to suffer from stress and burden in their work. Unfortunately, these professionals usually have less training or advice, hence the need for professional training in the field. Caregivers who are on the front line with the patient must have emotional health and adequate resources and strategies to work. Thus, the caregiver must be physical and psychologically healthy to be able to provide quality care (Arias et al., 2017; Losada et al., 2006). Formal caregivers recognise the need for and are interested in receiving training specific to caring for persons living with dementia (Breen et al., 2021; Flöjt et al., 2014).

They perform tasks such as offering support, administering medications, helping patients in intimate moments (such as bathing or dressing them if necessary), accompanying them with chores, offering them meals, and so on (Cancer.Net Editorial Board, 2012). Therefore, resilience can help them to cope with these potentially stressful situations, and aid in alleviating damage to their physical or psychological health (Fernández‐Lansac et al., 2012).

Future lines of research derive from the results obtained in this study. One possible line of research could be improving caregivers' skills in assistance and community services. Hence, new studies could study other variables related to care such as personality traits, empathy or emotional intelligence and the implementation and evaluation of training programs for professional caregivers.

Finally, among the limitations of the present work, the sample size is pointed out, as it might be higher to obtain greater statistical power and prediction among the variables.

As we have seen, the role of the caregiver is important in different areas (personal, familiar, social and communitarian). Moreover, community services for elderly people's care can be taken into consideration, which may invest in their patients' and workers' well‐being and their necessity of having a social support network, as it acts as a stress protective factor (Fernández‐Lansac & Crespo, 2011).

Resilience has been proven to be a protective factor for adaptation and against stress and burden among caregivers (Cejalvo et al., 2021; Palacio et al., 2020). Teaching caregivers how to develop a resilient style is considered a necessary subject in intervention programs. This will lead to a create healthier coping strategies, which will, in turn, improve the caregivers' quality of life.

## CONFLICT OF INTEREST

All of the authors declare they have no competing interests.

## Data Availability

Data available on request due to privacy/ethical restrictions.

## References

[hsc14110-bib-0001] Abellán, A. , Ayala, A. , Pérez, J. , & Pujol, R. (2018). Un perfil de las personas mayores en España, 2018. Indicadores estadísticos básicos (Vol. 17). Informes Envejecimiento en red. http://digital.csic.es/handle/10261/164389

[hsc14110-bib-0002] Abraha, I. , Rimland, J. M. , Trotta, F. M. , Dell'Aquila, G. , Cruz‐Jentoft, A. , Petrovic, M. , Gudmundsson, A. , Soiza, R. , O'Mahony, D. , Guaita, A. , & Cherubini, A. (2017). Systematic review of systematic reviews of non‐pharmacological interventions to treat behavioural disturbances in older patients with dementia. BMJ Open, 7(3), e012759. 10.1136/bmjopen-2016-012759 PMC5372076 28302633

[hsc14110-bib-0003] Arias, S. , Saavedra, F. J. , & Avilés, I. (2017). El cuidado, una actividad de riesgo en tiempos de crisis: Una revisión de la investigación con cuidadoras españolas. Psicoperspectivas, 16(1), 42–54. 10.5027/psicoperspectivas-vol15-issue3-full-796

[hsc14110-bib-0004] Auné, S. E. , Blum, D. , Abal, J. P. , Lozzia, G. S. , & Attorresi, H. F. (2014). La conducta prosocial: Estado actual de la investigación. Perspectivas en Psicología: Revista de Psicología y Ciencias Afines, 11(2), 21–33. http://www.redalyc.org/html/4835/483547666003/

[hsc14110-bib-0005] Becoña, E. B. (2006). Resiliencia: definición, características y utilidad del concepto. Revista de psicopatología y psicología clínica, 11(3), 125–146. 10.5944/rppc.vol.11.num.3.2006.4024

[hsc14110-bib-0107] Breen, L. J. , Johnson, A. R. , O'Connor, M. , Howting, D. , & Aoun, S. M. (2021). Challenges in palliative care research on family caregivers: Who volunteers for interviews? Journal of Palliative Medicine, 24(1), 112–115. 10.1089/jpm.2019.0672 32255736

[hsc14110-bib-0007] Burgueño, A . (2008). Atar para Cuidar: Uso de Sujeciones Físicas y Químicas en Personas Mayores dependientes que reciben cuidados prolongados . https://www.navarra.es/NR/rdonlyres/DB50F783‐BEE9‐494D‐A661‐7F7A50AC6302/107054/usodesujecionesenpersonasmayores1.pdf

[hsc14110-bib-0008] Cancer.Net Editorial Board . (2012). Conceptos básicos sobre el cuidado de un paciente. American Society of Clinical Oncology (ASCO): Cancer.net Articles . http://www.cancer.net/cancernet‐en‐español/asimilación/cuidado‐del‐paciente/ser‐cuidador‐de‐un‐paciente

[hsc14110-bib-0009] Caprara, G. , Steca, P. , Zelli, A. , & Capanna, C. A. (2005). New scale for measuring adults' prosocialness. European Journal of Psychological Assessment, 21(2), 77–89. 10.1027/1015-5759.21.2.77

[hsc14110-bib-0010] Cejalvo, E. , Martí‐Vilar, M. , Merino‐Soto, C. , & Aguirre‐Morales, M. T. (2021). Caregiving role and psychosocial and individual factors: A systematic review. Healthcare, 2021(9), 1690. 10.3390/healthcare9121690 PMC8700856 34946416

[hsc14110-bib-0011] Cerejeira, J. , Lagarto, L. , & Mukaetova‐Ladinska, E. B. (2012). Behavioral and psychological symptoms of dementia. Frontiers in Neurology, 3(73), 1–21. 10.3389/fneur.2012.00073 22586419 PMC3345875

[hsc14110-bib-0012] Cohen‐Mansfield, J. , Dakheel‐Ali, M. , Marx, M. S. , Thein, K. , & Regier, N. G. (2015). Which unmet needs contribute to behavior problems in persons with advanced dementia? Psychiatry Researh, 228, 59–64. 10.1016/j.psychres.2015.03.043 PMC4451402 25933478

[hsc14110-bib-0013] Connor, K. M. , & Davidson, J. R. T. (2003). Development of a new resilience scale: The Connor‐Davidson Resilience Scale (CD‐RISC). Depression and Anxiety, 18(2), 76–82. 10.1002/da.10113 12964174

[hsc14110-bib-0014] Crespo, M. , Ovejero, A. , Gómez, L. , Martínez, M. , & González, V. (2010). Factores de resiliencia ante el acaso y el abuso psicológico. Dialnet.

[hsc14110-bib-0015] Escotorin, P. , & Cirera, M. (2013). Salut i humanisme (2). Per què formar‐se en prosocialitat: noves vies per optimitzar la salut dels professionals sociosanitaris. Annals de Medicina, 96(3), 115–117. http://www.acmcb.es/files/495‐18‐FITXER/Annals_de_Medicina_96_3_2013.pdf

[hsc14110-bib-0016] Farran, C. J. , Gilley, D. W. , McCann, J. J. , Bienias, J. L. , Lindeman, D. A. , & Evans, D. A. (2007). Efficacy of behavioral interventions for dementia caregivers. Western Journal of Nursing Research, 29(8), 944–960. 10.1177/0193945907303084 17596639

[hsc14110-bib-0017] Fauth, E. B. , & Gibbons, A. (2014). Which behavioral and psychological symptoms of dementia are the most problematic? Variability by prevalence, intensity, distress ratings, and associations with caregiver depressive symptoms. International Journal of Geriatric Psychiatry, 29(3), 263–271. 10.1002/gps.4002 23846797

[hsc14110-bib-0018] Fernández‐Lansac, V. , & Crespo, M. (2011). Resiliencia, Personalidad Resistente y Crecimiento en Cuidadores de Personas con Demencia en el Entorno Familiar: Una Revisión. Clínica y Salud, 22(1), 21–40. 10.5093/cl2011v22n1a2

[hsc14110-bib-0019] Fernández‐Lansac, V. , López, M. C. , Cáceres, R. , & Rodríguez‐Poyo, M. (2012). Resiliencia en cuidadores de personas con demencia: estudio preliminar. Revista Española de Geriatría y Gerontología, 47(3), 102–109. 10.1016/j.regg.2011.11.004 22579610

[hsc14110-bib-0108] Flöjt, J. , Hir, U. L. , & Rosengren, K. (2014). Need for preparedness: Nurses’ experiences of competence in home health care. Home Health Care Management & Practice, 26(4), 223–229. 10.1177/1084822314527

[hsc14110-bib-0020] Flores, N. , Jenaro, C. , Moro, L. , & Tomsa, R. (2014). Health and quality of life of family caregivers and professionals of dependent elderly people: A comparative study. Salud y calidad de Vida de cuidadores familiares y profesionales de personas mayores dependientes: Estudio comparativo. European Journal of Investigation in Health Psychology and Education., 4(2), 79–88. 10.1989/ejihpe.v4i2.55

[hsc14110-bib-0021] Forján, R. , & Morelatto, G. (2018). Estudio comparativo de factores de resiliencia en docentes de contextos socialmente vulnerables. Psicogente, 21(40), 277–296. 10.17081/psico.21.40.3075

[hsc14110-bib-0103] García, L. (2014). Resiliencia y personalidad en el personal de urgencias hospitalarias y Extrahospitalarias del principado de Asturias [Thesis in internet]. Universidad de Oviedo, Facultad de Ciencias de la Salud. http://digibuo.uniovi.es/dspace/handle/10651/27807

[hsc14110-bib-0022] García, L. (2016). Resiliencia y personalidad en el personal de urgencias hospitalarias y Extrahospitalarias del principado de Asturias (tesis de grado en internet) Oviedo: Universidad de Oviedo. Facultad de Ciencias de la Salud. http://digibuo.uniovi.es/dspace/handle/10651/27807

[hsc14110-bib-0023] Gaugler, J. E. , Kane, R. L. , & Newcomer, R. (2007). Resilience and transitions from dementia caregiving. The Journals of Gerontology; Series B, 62(1), 38–44. 10.1093/geronb/62.1.p38 17284556

[hsc14110-bib-0024] González, E. , & Domínguez, M. L. (2000). Factores que inciden en la actuación profesional con personas mayores. Papeles del Psicólogo, 76, 9–12. https://www.researchgate.net/publication/28070082_Factores_que_inciden_en_la_actuacion_profesional_con_personas_mayores

[hsc14110-bib-0025] González, N. , Valdez, J. , & Zavala, Y. (2008). Resiliencia en adolescentes mexicanos. Enseñanza e Investigación en psicología, 13(1), 41–52. https://www.researchgate.net/publication/40441109_Resiliencia_en_adolescentes_mexicanos

[hsc14110-bib-0026] González‐Arratia, N. I. , & Valdez, J. L. (2013). Resiliencia: Diferencias por Edad en Hombres y Mujeres Mexicanos. Acta de Investigación Psicológica, 3(1), 941–955. http://www.psicologia.unam.mx/pagina/es/155/acta‐de‐investigacion‐psicologica

[hsc14110-bib-0027] Huang, M. F. , Huang, W. H. , Su, Y. C. , Hou, S. Y. , Chen, H. M. , Yeh, Y. C. , & Chen, C. S. (2015). Coping strategy and caregiver burden among caregivers of patients with dementia. American Journal of Alzheimer's Disease and Other Dementias, 30(7), 694–698. 10.1177/1533317513494446 PMC10852617 23813690

[hsc14110-bib-0101] INE . (2020). Anuario Estadístico de España. INE. http://www.ine.es/prodyser/pubweb/anuarios_mnu.htm

[hsc14110-bib-0029] Ismail, Z. , Smith, E. E. , Geda, Y. , Sultzer, D. , Brodaty, H. , Smith, G. Y. , Agúera‐Ortiz, L. , Sweet, R. , Miller, D. , & Lyketsos, C. G. (2016). Neuropsychiatric symptoms as early manifestations of emergent dementia: Provisional diagnostic criteria for mild behavioral impairment. Alzheimer's & Dementia, 12, 195–202. 10.1016/j.jalz.2015.05.017 PMC4684483 26096665

[hsc14110-bib-0030] Izal, M. , Losada, A. , Marquez, M. , & Montorio, I. (2003). Analysis of general practitioners' and social workers' perception of their training to attend caregivers of the dependent elderly. Revista Española de Geriatría y Gerontología, 38(4), 203–211. 10.1016/S0211-139X(03)74885-9

[hsc14110-bib-0031] Judkins, S. K. (2001). Stress, hardiness, and coping strategies among midlevel nurse managers: Implications for continuing higher education. Dissertation Abstracts International, 63(129), 6. https://pdfs.semanticscholar.org/ccce/6c947d0ac0f301a6a1cc0b16259e63e53529.pdf

[hsc14110-bib-0032] Kales, H. C. , Gitlin, L. N. , & Lyketsos, C. G. (2015). Assessment and management of behavioral and psychological symptoms of dementia. British Medical Journal, 350, h369. 10.1136/bmj.h369 25731881 PMC4707529

[hsc14110-bib-0034] Kong, E. H. , Choi, H. , & Evans, L. K. (2017). Staff perceptions of barriers to physical restraint‐reduction in long‐term care: A meta‐synthesis. Journal of Clinical Nursing, 26(1–2), 49–60. 10.1111/jocn.13418 27270849

[hsc14110-bib-0035] Koopman, E. , Heemskerk, M. , van der Beek, A. J. , & Coenen, P. (2020). Factors associated with caregiver burden among adult (19–64 years) informal caregivers–an analysis from Dutch municipal health service data. Health & Social Care in the Community, 28(5), 1578–1589. 10.1111/hsc.12982 32207221 PMC7496310

[hsc14110-bib-0036] Lago, R. , & Alós, P. (2012). Estudio descriptivo sobre el perfil de los cuidadores de personas con demencia: la feminización del cuidado. Psicogente, 15(27), 24–35. http://www.redalyc.org/articulo.oa?id=497552360004

[hsc14110-bib-0037] Losada, A. , Márquez, M. , Vara‐García, C. , Gallego, L. , Romero, R. , & Olazarán, J. (2017). Impacto psicológico de las demencias en las familias: propuesta de un modelo integrador. Revista Clínica Contemporánea, 8, 1–27. 10.5093/cc2017a4

[hsc14110-bib-0038] Losada, A. , Montorio, I. , Izal, M. , & Márquez, M. (2006). Estudio e intervención sobre el malestar psicológico de los cuidadores de personas con demencia. El papel de los pensamientos disfuncionales. IMSERSO. http://ibdigital.uib.es/greenstone/collect/portal_social/import/msan/msan0082.pdf

[hsc14110-bib-0039] Martín, R. A. (2015). Estrés laboral en Enfermería: La escasez de personal actual en cuidados intensivos. Revista Enfermería del Trabajo, 5(3), 76–81. https://dialnet.unirioja.es/servlet/articulo?codigo=5213011

[hsc14110-bib-0041] Martí‐Vilar, M. , & Navarro, R. (2017). Lo que dice la ciencia sobre la empatía y algunos cuestionarios útiles. In E. R. Roche & P. Escotorin (Eds.), Cuidar con actitud prosocial. Nuevas propuestas para cuidadores (pp. 141–170). Ciudad Nueva.

[hsc14110-bib-0042] Martorell, C. , González, R. , Ordoñez, A. , & Gómez, O. (2011). Estudio confirmatorio del Cuestionario de Conducta Prosocial (CEP) y su relación con variables de personalidad y socialización. Revista Iberoamericana de Diagnóstico y Evaluación Psicológica, 32(2), 35–52. https://www.redalyc.org/pdf/4596/459645440003.pdf

[hsc14110-bib-0043] Menezes de Lucena, V. , Fernández‐Calvo, B. , Hernández, L. M. , Ramos, F. C. , & Contador, I. C. (2006). Resilience and the burnout‐engagement model in formal caregivers of the elderly. Psicothema, 18(4), 791–796. http://www.psicothema.com/psicothema.asp?id=3310 17296119

[hsc14110-bib-0044] Mollart, L. , Skinner, V. M. , Newing, C. , & Foureur, M. (2013). Factors that may influence midwives work‐related stress and burnout. Women and Birth, 26(1), 26–32. 10.1016/j.wombi.2011.08.002 21889431

[hsc14110-bib-0045] Moore, K. , Ozanne, E. , Ames, D. , & Dow, B. (2013). How do family carers respond to behavioral and psychological symptoms of dementia? International Psychogeriatrics, 25(5), 743–753. 10.1017/S1041610213000070 23425394

[hsc14110-bib-0046] Notario‐Pacheco, B. , Solera‐Martínez, M. , Serrano‐Parra, M. D. , Bartolomé‐Gutiérrez, R. , García‐Campayo, J. , & Martínez‐Vizcaíno, V. (2011). Reliability and validity of the Spanish version of the 10‐item Connor‐Davidson Resilience Scale (10‐item CD‐RISC) in young adults. Health and Quality of Life Outcomes, 9(1), 63. 10.1186/1477-7525-9-63 21819555 PMC3173284

[hsc14110-bib-0047] Ornstein, K. , Gaugler, J. E. , Devanand, D. P. , Scarmeas, N. , Zhu, C. , & Stern, Y. (2013). The differential impact of unique behavioral and psychological symptoms for the dementia caregiver: How and why do patients' individual symptom clusters impact caregiver depressive symptoms? American Journal of Geriatric Psychiatry, 21, 1277–1286. 10.1016/j.jagp.2013.01.062 PMC3543497 24206939

[hsc14110-bib-0048] Palacio, G. C. , Krikorian, A. , Gómez‐Romero, M. J. , & Limonero, J. T. (2020). Resilience in caregivers: A systematic review. American Journal of Hospice and Palliative Medicine, 37(8), 648–658. 10.1177/1049909119893977 31830813

[hsc14110-bib-0049] Plaza‐Carmona, M. , & Requena‐Hernández, C. (2016). Uso de la comunicación prosocial en profesionales sanitarios y sociales con personas mayores: Estudio piloto. Gerokomos, 27(1), 13–18. http://scielo.isciii.es/pdf/geroko/v27n1/04_originales_03.pdf

[hsc14110-bib-0050] Ramírez, M. T. G. , & Hernández, R. L. (2008). Síntomas psicosomáticos y estrés: comparación de un modelo estructural entre hombres y mujeres. Ciencia‐Uanl, 11(4), 11. https://dialnet.unirioja.es/servlet/articulo?codigo=2865054

[hsc14110-bib-0051] Reymond, A. G. , Gispert, E. A. , Llibre, J. J. , Castell‐Florit, P. , & Zayas, T. (2019). Percepción de las demencias y de la intersectoralidad en el contexto del Policlínico Docente Playa. Revista Cubana Salud Pública, 1(45), e1128. https://www.scielosp.org/article/rcsp/2019.v45n1/e1128/es/

[hsc14110-bib-0052] Roche, R. , & Escotorin, P. (2017). Cuidar con actitud prosocial. Nuevas propuestas para cuidadores. Ciudad Nueva.

[hsc14110-bib-0053] Rodríguez, A. , & Ortunio, M. S. (2019). Resiliencia en trabajadores de la salud de una unidad de cuidados intensivos pediátricos. Revista Venezolana de Salud Pública, 7(1), 27–33. http://portal.amelica.org/ameli/jatsRepo/234/234990004/html/

[hsc14110-bib-0054] Rodríguez, C. (2015). Economía feminista y economía del cuidado. Nueva Sociedad, 256, 30–44. http://hdl.handle.net/11336/47084

[hsc14110-bib-0055] Rook, C. , Smith, L. , Johnstone, J. , Rossato, C. , Sánchez, G. F. L. , Suárez, A. D. , & Roberts, J. (2018). Reconceptualising workplace resilience—A cross‐disciplinary perspective. Anales de Psicología/Annals of Psychology, 34(2), 332–339. https://apps.webofknowledge.com/full_record.do?product=UA&search_mode=GeneralSearch&qid=7&SID=D26JKZtPVwmsBsHGIfc&page=3&doc=29

[hsc14110-bib-0105] Ruiz‐Robledillo, N. , & Moya‐Albiol, L. (2012). El cuidado informal: Una visión actual. Revista de motivación y emoción, 1(1), 22–30. http://reme.uji.es/reme/3‐albiol_pp_22‐30.pdf

[hsc14110-bib-0104] Sarabia‐Cobo, C. M. , Pérez, V. , de Lorena, P. , Domínguez, E. , Hermosilla, C. , Nuñez, M. J. , Vigueiro, M. , & Rodríguez, L. (2016). The incidence and prognostic implications of dysphagia in elderly patients institutionalized: A multicenter study in Spain. Applied Nursing Research, 30, e6–e9. 10.1016/j.apnr.2015.07.001 26235494

[hsc14110-bib-0056] Sanz, A. I. , Iriarte, S. , & Gascón, A. (2016). Aspectos sociodemográficos y laborales en el error asistencial de enfermería. Enfermería Global, 15(43), 176–188. http://scielo.isciii.es/pdf/eg/v15n43/docencia2.pdf

[hsc14110-bib-0102] Tan, Z. S. , Ramirez, K. , Soh, M. , & Ercoli, L. (2017). The effects of an intensive dementia caregiver training course on caregiver knowledge and perceived competence. Alzheimer's & Dementia, 13(7), 491. 10.1016/j.jalz.2017.06.533

[hsc14110-bib-0059] Taycan, O. , Erdoğan Taycan, S. , & Çelik, C. (2013). El impacto del servicio de salud obligatorio en los médicos y el agotamiento en una provincia del este de Anatolia. Diario turco de psiquiatría, 24, 182–191.

[hsc14110-bib-0060] Teri, L. , Huda, P. , Gibbons, L. , Young, H. , & van Leynseele, J. (2005). STAR: A dementia‐specific training program for staff in assisted living residences. The Gerontologist, 45(5), 686–693. 10.1093/geront/45.5.686 16199404

[hsc14110-bib-0106] The jamovi project . (2022). *jamovi (Version 2.3)* [Computer Software]. https://www.jamovi.org

[hsc14110-bib-0061] Torres, J. (2008). Aspectos psicológicos en cuidadores de formales de ancianos: carga y afrontamiento del estrés (un estudio en población sociosanitaria) (Tesis doctoral inédita). Universidad de Huelva. http://rabida.uhu.es/dspace/handle/10272/2645

[hsc14110-bib-0063] Wilks, S. , & Vonk, M. (2008). Private prayer among Alzheimer's caregivers: Mediating burden and resiliency. Journal of Gerontological Social Work, 50, 113–131. 18510194 10.1300/J083v50n3_09

[hsc14110-bib-0064] World Health Organization . (2018). Dementia . http://www.who.int/mediacentre/factsheets/fs362/en/

[hsc14110-bib-0065] Yıldızhan, E. , Ören, N. , Erdoğan, A. , & Bal, F. (2019). The burden of care and burnout in individuals caring for patients with Alzheimer's disease. Community Mental Health Journal, 55, 304–310. 10.1007/s10597-018-0276-2 29680976

